# Quarterly Report of Diseases Treated in the St. George's and St. James's General Dispensary; from July 13 to October 12, 1822

**Published:** 1822-11

**Authors:** George Gregory

**Affiliations:** Senior Physician to the Dispensary, &c. &c.


### STATISTICAL MEDICINE.

Art. I.—*Quarterly Report of Diseases treated in the St. George's and St. James's General Dispensary; from July* 13 *to October* 12, 1822. By George Gregory, m.d. Senior Physician to the Dispensary, &c. &c.

| | | | |
|---|---|---|---|
| Acute Constitutional Affections | Common Continued Fever | 30 | |
| | Fever with predominant Bilious Symptoms | 40 | |
| | Typhus | 6 | |
| | Hectic Fever | 1 | |
| | Infantile Remittent Fever | 25 | |
| | Measles | 5 | |
| | Scarlatina and its sequelæ | 13 | |
| | Rheumatic Affections | 18 | 138 |
| Acute and Chronic Diseases of the Head | Phrenitis and Hydrocephalus | 5 | |
| | Palsy | 5 | |
| | Epilepsy | 2 | |
| | Vertigo | 3 | |
| | Pain of the Head | 10 | 25 |
| Acute and Chronic Diseases of the Thorax | Cynanche Tonsillaris | 3 | |
| | Pneumonia | 10 | |
| | Chronic Bronchitis | 20 | |
| | Hæmoptysis | 3 | |
| | Phthisis | 8 | |
| | Asthma | 5 | |
| | Dyspnœa | 2 | |
| | Pertussis | 11 | |
| | Palpitation | 2 | |
| | Pain of the Side | 1 | 65 |

| | | | |
|---|---|---|---|
| Diseases of the Chylopoietic Viscera.... | Abdominal Inflammation | 2 | |
| | Bilious (without Fever) | 12 | |
| | Disordered Bowels of Infants | 14 | |
| | Dyspepsia | 54 | |
| | Hæmatemesis | 1 | |
| | Chronic Hepatitis | 5 | |
| | Colic | 6 | |
| | Diarrhœa | 6 | |
| | Cholera | 1 | |
| | Jaundice | 2 | |
| | Mesenteric Obstruction | 1 | |
| | Piles | 6 | |
| | Diseased Rectum | 1 | |
| | Worms | 2— | 113 |
| Diseases of the Urinary and Uterine Systems.. | Paralysis of the Biadder | 1 | |
| | Nephralgia | 1 | |
| | Menorrhagia | 2 | |
| | Amenorrhœa | 10 | |
| | Hysteria | 3 | |
| | Dysuria | 1 | |
| | Prolapsus Uteri | 3 | |
| | Carcinoma Uteri | 2 | |
| | Retention of Urine | 2 | |
| | Leuchorrœa | 3— | 28 |
| Chronic Constitutional Affections .......... | Dropsy | 3 | |
| | Scrofula | 2 | |
| | Debility | 8 | |
| | Rickets | 1 | |
| | Chronic Aphtha | 1 | |
| | Climacteric | 2 | |
| | Plethora | 1 | |
| | Hypochondriasis | 2 | |
| | Secondary Syphilis | 5— | 25 |
| Diseases of the Skin.... | Strophalus | 1 | |
| | Prurigo | 1 | |
| | Lepra | 1 | |
| | Psoriasis | 3 | |
| | Urticaria | 1 | |
| | Purpura Urticans | 1 | |
| | Impetigo | 1 | |
| | Porrigo Favosa | 7 | |
| | Ecthyma Cachecticum | 1 | |
| | Scabies | 2 | |
| | Herpes | 1 | |
| | Herpes Zoster | 2 | |
| | Eruptions not well characterized | 12— | 34 |
| Diseases strictly Local .. | Fractures, Luxations, and severe Accidents | 6 | |
| | Sprains, Bruises, Scalds, and slight Wounds | 38 | |
| | Local Inflammations | 10 | |
| | Ophthalmia | 4 | |
| | Tumors | 6 | |
| | Ulcers of the Leg | 17 | |
| | Ulcers of the Penis | 6 | |
| | Bubo | 5 | |
| | Gonorrhœa | 7 | |
| | Hernia Humoralis | 1 | |
| | Stricture | 1 | |
| | Dropsical Bursæ | 1 | |
| | Abscesses | 13 | |
| | Hernia | 1 | |
| | Node on the Tibia | 1— | 117 |
| Cases not reported | | 29— | 29 |
| | Total | | 574 |

The summer and autumn of 1822 will long live in the recollection of those to whom the weather is an object of daily solicitude. It is many years since the inhabitants of London have enjoyed so long, and so uninterrupted, a series of fine weather. In comparison, indeed, of the preceding year, we may almost be said to have been living under an Italian sky.

It is a curious, but well ascertained, fact, that these *open* seasons are not necessarily healthy, and the experience of the present year fully corroborates this currently received opinion. Sickness has been very general all over London, and its character has been well marked and uniform. A survey of the annexed table of admissions into the St. George's and St. James's Dispensary will point out the prevalent diseases of the last three months; which a slight comment may, perhaps, tend still further to illustrate.

Fever has predominated over every other form of disease, and it has principally shown itself under the following characters:

1. Throughout the hottest part of July and August, *bilious fever* was constantly met with. Purgative medicines were indispensable in the cure of it, and it seldom resisted the continued and judicious administration of them. 2. Early in September, the fever assumed all the characters of the *low nervous* kind, as described by the old authors. It was attended with very considerable irritability of the stomach and bowels, rendering it necessary to be extremely cautious in the administration of purgative medicines. It was very often aggravated by calomel and antimony, which in former seasons have proved so serviceable: castor oil, on the other hand, seemed to agree with almost every patient. General blood-letting was hardly ever required, and, where attempted, was seldom of any use. Two or three leeches to the temples relieved the accompanying head-ach, with a degree of quickness and *certainty*, which it is very difficult to account for, if we confine our view to the mere loss of blood which they occasion. This form of fever was remarkable for the great degree of languor which accompanied it, and the tedious convalescence by which it was followed. It was often necessary to support the patient with broths during its continuance, and the aromatic bitters contributed materially, in its latter stages, to renovate the tone of the stomach.—3. Some of the severer cases of fever put on all the characters of genuine typhus. These were, for the most part, sent to the London Fever Hospital; and I am glad to have an opportunity of expressing publicly the sense which I entertain, in common with many other practitioners, of the value of that excellent institution. That value is greatly enhanced by the attention which is paid to the *instant* removal of the patient.—4. Children have particularly suffered, during the last quarter, from a *remitting* form of fever, and it has proved in many instances fatal.—5. Scarlet fever and hooping-cough have been more than usually prevalent, but, for the most part, of a mild character.

In fact, it is rather the *extent* of febrile affection, than its *severity*, which has distinguished the medical history of the last three months. No acute inflammations, of any marked character, have been met with; and, in particular, acute rheumatism has been very uncommon. The lancet has been, therefore, but very little in requisition; and, among those who were bled, I did not observe *buffy* blood in more than two or three instances.

It is hardly possible to avoid drawing a conclusion from these facts in reference to the impossibility of judging of the treatment proper in *one* epidemic, from that which was successfully pursued in another. Those who advocate the propriety of bleeding in typhoid fevers *generally*, and without reference to the character of the epidemic, (if, indeed, there are any such,) would have received an instructive lesson from studying disease as it has appeared in London during the last three months.

Among the chronic diseases of this period, dyspepsia stands, as usual, pre-eminent. I have been much more successful in the treatment of this disorder since I gave up the employment of bitters, absorbents, tonics, and of active purgative draughts, and trusted mainly to the exhibition (twice in the week) of a mixture of four grains of calomel with four of antimonial powder, made up into two pills. This combination appears to be particularly well adapted to clear the stomach of that load of thickened and depraved mucus which adheres to its internal coat, and prevents the food from coming in contact with it. That this is the real state of the stomach in most of the genuine cases of dyspepsia which are met with in dispensary practice, I am fully persuaded.

The remaining diseases of this quarter present no features of any particular interest.

The fatal cases amounted to seven,—viz. one Remittent Fever, two Phthisis, one Scrofula, one Scarlatina, one Pneumonia, and one Bronchitis Chronica; but, in those which were examined after death, nothing was observed which is in any way worthy of separate notice.

